# Physicochemical Properties of Glycine-Based Ionic Liquid [QuatGly-OEt][EtOSO_3_] (2-Ethoxy-1-ethyl-1,1-dimethyl-2-oxoethanaminium ethyl sulfate) and Its Binary Mixtures with Poly(ethylene glycol) (M_w_ = 200) at Various Temperatures

**DOI:** 10.3390/ijms12128750

**Published:** 2011-12-02

**Authors:** Tzi-Yi Wu, Bor-Kuan Chen, Lin Hao, Yuan-Chung Lin, H. Paul Wang, Chung-Wen Kuo, I-Wen Sun

**Affiliations:** 1 Department of Chemistry, National Cheng Kung University, Tainan 70101, Taiwan; E-Mails: t718z@yahoo.com.tw (T.-Y.W); t0322627@seed.net.tw (L.H.); 2 Department of Materials Engineering, Kun Shan University, Tainan 71003, Taiwan; E-Mail: chenbk@mail.ksu.edu.tw; 3 Institute of Environmental Engineering, National Sun Yat-Sen University, Kaohsiung 804, Taiwan; E-Mail: yclin@faculty.nsysu.edu.tw; 4 Department of Environmental Engineering, National Cheng Kung University, Tainan 70101, Taiwan; E-Mail: wanghp@mail.ncku.edu.tw; 5 Department of Chemical and Materials Engineering, National Kaohsiung University of Applied Sciences, Kaohsiung 807, Taiwan; E-Mail: welly@cc.kuas.edu.tw

**Keywords:** ionic liquids, density, viscosity, refractive index, excess molar volume, conductivity

## Abstract

This work includes specific basic characterization of synthesized glycine-based Ionic Liquid (IL) [QuatGly-OEt][EtOSO_3_] by NMR, elementary analysis and water content. Thermophysical properties such as density, ρ, viscosity, η, refractive index, *n*, and conductivity, κ, for the binary mixture of [QuatGly-OEt][EtOSO_3_] with poly(ethylene glycol) (PEG) [M_w_ = 200] are measured over the whole composition range. The temperature dependence of density and dynamic viscosity for neat [QuatGly-OEt][EtOSO_3_] and its binary mixture can be described by an empirical polynomial equation and by the Vogel-Tammann-Fucher (VTF) equation, respectively. The thermal expansion coefficient of the ILs is ascertained using the experimental density results, and the excess volume expansivity is evaluated. The negative values of excess molar volume for the mixture indicate the ion-dipole interactions and packing between IL and PEG oligomer. The results of binary excess property (*V*_m_^E^ ) and deviations (Δη, Δ*_x_n*, Δ*_Ψ_n*, Δ*_x_R*, and Δ*_Ψ_R*) are discussed in terms of molecular interactions and molecular structures in the binary mixture.

## 1. Introduction

Ionic liquids (ILs) are commonly defined as organic and inorganic salts that have melting temperatures lower than 373 K [1]. In recent years, ILs have attracted increasing attention and intensive investigation and witnessed a steady growth from the academics to industry due to their distinctive properties, such as wide temperature range of application, high thermal stability [2,3], nonflammability, wide electrochemical window [4,5], high electrical conductivity [6,7], and highly favorable solubility of metal oxides [8]. They have been used in a broad variety of extraction [9], separation [10] and biotechnology [11,12], especially as promising “green” electrolyte for electrochemical devices such as dye-sensitized solar cells [13–15], lithium ion batteries [16,17], super capacitors, and fuel cells [18,19]. ILs can be tuned by varying the combination of various cations and anions [20]. So far, ILs with cations derived from imidazolium, pyridinium, quaternary ammonium, and pyrrolidinium have been extensively studied [21,22]. The development of bio-renewable ILs based on amino acids and their derivatives to replace the above cations is another promising approach because amino acids and their derivatives are the most abundant natural source of quaternary nitrogen precursors [23]. Amino acid ionic liquids (AAILs) were first prepared by coupling various amino acids anions with suitable cations [24], and then many amino acid-based ILs were prepared including amino acids as anions [25], amino acids as cations [26] or amino acid derivatives [27]. Until now, the viscosity of AAILs are 1 to 3 orders of magnitude greater than those of traditional organic liquids, which affect the mass transfer rate, leading to much more power needed for mixing in liquid reactions or separation processes. In some current studies, the problem of high viscosity can be resolved partially by means of adding organic solvents to pure ionic liquid [28]. Although the addition of organic solvents into ILs decreases the viscosity of mixture significantly, the conventional organic solvents have high volatility. Alternatively, some scientists studied the thermodynamic properties of ethylene glycol derivatives. Poly(ethylene glycol) (PEG) is one of the ethylene glycol derivatives, PEG of various molecular weights have been widely used in processes across many industrial sectors, as a result of being non-toxic, biodegradable, inexpensive, widely available, and a very low volatility [29,30]. Low molecular weight PEG (M_w_ = 200) is liquid at room temperature, making it easy to combine with ILs, generate solvent systems for use in advanced, environmentally friendly processes [30]. Furthermore, solvent systems composed of (IL + PEG) provide exciting “hybrid green” systems that may afford interesting and favorably modified physicochemical properties enhancing the potential of both ILs and PEGs in many chemical applications, such as the addition of PEG200 in ionic liquid enhanced the kinetics of absorption and desorption of CO_2_ significantly [29], unusual fluorescence probe behavior of pyrene in IL + PEG hybrid systems [31], the use of PEG-600 as cosolvent of the ILs ([C_4_mim][BF_4_] or [C_4_mim][PF_6_], in the presence of NaBH_4_ and at 180–200 °C, the morphology of the nanostructures of Se and Te is obtained [32].

In this paper, we evaluate the density, viscosity, conductivity, and refractive index of the bio-renewable glycine-based IL [QuatGly-OEt][EtOSO_3_] and its binary mixture with PEG200 over the whole composition range and temperatures between (293.15 and 353.15) K at ambient pressure. From these experimental results, excess molar volume, viscosity deviations, and refractive index deviations from the ideal behavior were characterized. The excess properties were calculated and then correlated, at each temperature, as a function of composition by a Redlich-Kister-type equation. The impact of the nature of the solute on the thermodynamic properties of binary mixture was investigated in detail.

## 2. Results and Discussion

### 2.1. Density and Excess Molar Volume

**Figure 1:**
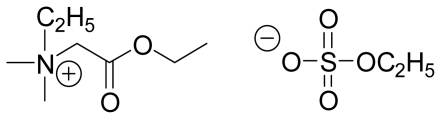
Molecular structure of 2-ethoxy-1-ethyl-1,1-dimethyl-2-oxoethanaminium ethyl sulfate ([QuatGly-OEt][EtOSO_3_]).

The densities of neat IL [QuatGly-OEt][EtOSO_3_], neat PEG200, and {PEG200 (1) + [QuatGly-OEt][EtOSO_3_] (2)} binary mixture over the temperature range (293.15 to 353.15) K and at atmospheric pressure are presented in Table 1, and the structure of IL is shown in Figure 1. From analysis of the data, it was found that the densities depend more strongly on the mole fraction of PEG200 in the mixture than on temperature; the density trend for the binary system with respect to temperature decreased with increasing temperature.

**Figure 2:**
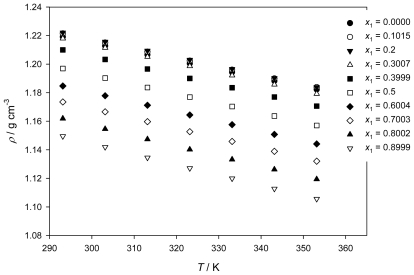
Density ρ of the {PEG200 (1) + [QuatGly-OEt][EtOSO_3_]} binary system as a function of temperature at various mole fractions of the PEG200.

As can be seen from Table 1 and Figure 2, the density decreases with increasing temperature even though the variation is very small. If the variation of the density with the temperature is considered as linear, then the change in the calculated thermal expansion coefficient values becomes nearly constant. In order to have an improved accuracy, a second order polynomial of the following form was used to correlate the variation of the density with the temperature for {PEG200 (1) + [QuatGly-OEt][EtOSO_3_] (2)} binary system studied in the present work.

(1) $$\rho =b_{0}+b_{1}\cdot T+b_{2}\cdot T^{2}$$

where *T* is the temperature and *b*_0_, *b*_1_, and *b*_2_ are the correlation coefficients. The correlation coefficients were estimated using the least square method, and the values of the coefficients are listed in Table 2 together with the standard deviations (σ) estimated using the following equation:

(2) $$\sigma ={\left[\frac{\sum {\left(Z_{\text{exp}}-Z_{\text{cal}}\right)}^{2}}{n}\right]}^{1/2}$$

where *n* is the number of experimental points, and *Z*_exp_ and *Z*_cal_ are the experimental and calculated values, respectively.

**Figure 3:**
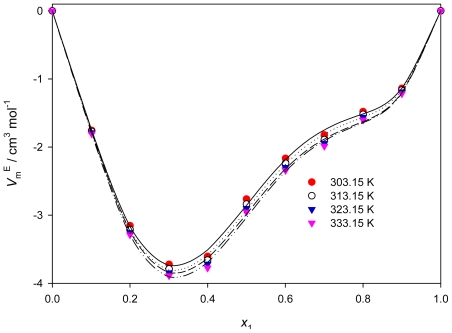
Excess molar volumes for the binary system {PEG200 (1) + [QuatGly-OEt][EtOSO_3_] (2)} and fitted curves using the Redlich-Kister parameters.

Excess thermodynamic properties are of great importance for understanding the nature of molecular interaction in binary mixtures. The excess molar volume, *V*_m_^E^, which is the difference between the real and ideal mixing behaviors, is defined by:

(3) $$V_{\text{m}}^{E}=V_{\text{m}}-x_{1}V_{1}^{o}-x_{2}V_{2}^{o}$$

where *V*_m_ represents the volume of a mixture containing one mole of (PEG200 + [QuatGly-OEt][EtOSO_3_]), *x*_1_ and *x*_2_ are the mole fractions of components 1 (PEG200) and 2 ([QuatGly-OEt][EtOSO_3_]), respectively, and *V*_1_^0^ and *V*_2_^0^ are the molar volumes of the pure components, respectively. The excess molar volumes of mixtures over the entire composition range were calculated from our measurements of density according to the following equation:

(4) $$V_{\text{m}}^{E}=\frac{x_{1}M_{1}+x_{2}M_{2}}{\rho }-\frac{x_{1}M_{1}}{\rho_{1}}-\frac{x_{2}M_{2}}{\rho_{2}}$$

where ρ_1_, ρ_2_, and ρ are the densities of neat PEG200, [QuatGly-OEt][EtOSO_3_], and their mixture, respectively; *M*_1_ and *M*_2_ are the molar masses of PEG200 and [QuatGly-OEt][EtOSO_3_], respectively. The *V*_m_^E^ values of mixtures are summarized in Table 1. The excess molar volumes of mixtures *versus* the mole fraction of PEG200 from 293.15 to 353.15 K are plotted in Figure 3. The excess molar volume of mixtures is mainly due to the variation in inter molecular forces between the compounds or the variation in the molecular packing due to the differences in the size and shape of the molecules forming a binary mixture with other compound. As shown in Figure 3, the excess molar volumes are asymmetric and negative for all of the systems studied over the entire composition range, the minimum of *V*_m_^E^ is reached at a mole fraction of PEG200 near *x*_1_ = 0.3, and the absolute values of the excess volume increase with increasing temperature. The negative excess molar volumes indicate that a more efficient packing and/or attractive interaction occurred when the ionic liquid and organic molecular liquids were mixed. The molar volume for [QuatGly-OEt][EtOSO_3_] is 234.07 cm^3^ mol^−1^, which is greater than the molar volumes of PEG200 (176.81 cm^3^ mol^−1^) at *T* = 293.15 K. The large difference between molar volumes of the molecular liquid and [QuatGly-OEt][EtOSO_3_] imply that it is possible that the relatively small organic molecules fit into the interstices upon mixing. Therefore, the filling effect of organic molecular liquids in the interstices of ionic liquids, and the ion-dipole interactions between organic molecular liquid and the glycine ester of the ionic liquids, all contribute to the negative values of the molar excess volumes. A similar phenomenon has been observed for ([bmim][PF_6_] + MeCN) and ([bmim][PF_6_] + MeOH) by Zafarani-Moattar and Shekaari [33].

### 2.2. Volume Expansivity and Excess Volume Expansivity

Density results for the ILs studied in current work were also used to derive other thermodynamic properties such as the coefficient of thermal expansion. From the density-temperature dependence, the volume expansivity (coefficient of thermal expansion), α, is defined as:

(5) $$\alpha =\frac{1}{V}{\left(\frac{\partial V}{\partial T}\right)}_{p}=-\frac{1}{\rho }{\left(\frac{\partial \rho }{\partial T}\right)}_{p}$$

where subscript *p* indicates constant pressure. The α values of pure [QuatGly-OEt][EtOSO_3_] and PEG200, and their mixture are summarized in Table 3. The values indicate that the thermal expansion coefficient of this binary mixture (PEG200 + [QuatGly-OEt][EtOSO_3_]) increases with increasing temperature and increases with increasing mole fraction of the PEG200. However, this trend also shows that the change in the thermal expansion coefficient with the temperature is not as significant as compared to the change with increasing mole fraction.

Next, the corresponding excess function was determined. The excess volume expansivity was calculated using:

(6) $$\alpha^{\text{E}}=\alpha -\phi_{1}^{\text{id}}\alpha_{1}-\phi_{2}^{\text{id}}\alpha_{2}$$

where *ϕ*_1_^id^ is an ideal volume fraction given by the following relation:

(7) $$\phi_{1}^{id}=\frac{x_{1}V_{\text{m}1}}{x_{1}V_{\text{m}1}+x_{2}V_{\text{m}2}}$$

where *V*_mi_ is for the molar volume of pure component *i*.

**Figure 4:**
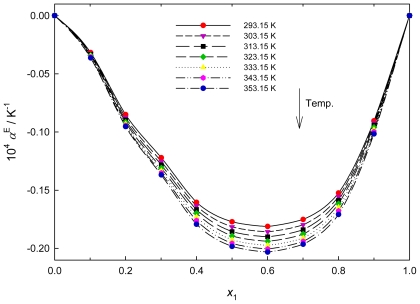
Plot of excess volume expansivity, α^E^, of the {PEG200 (1) + [QuatGly-OEt][EtOSO_3_] (2)} binary system *versus* mole fraction *x*_1_ at various temperatures.

Typical concentration dependencies of excess volume expansivity are given in Figure 4 for the {PEG200 + [QuatGly-OEt][EtOSO_3_]} binary system. The values of excess thermal coefficient α^E^ are found to be negative over the whole composition range for the binary system at all the investigated temperatures. The curves are asymmetrical, with the minimum located at the IL mole fraction of about 0.6. In general, negative α^E^ values indicate the presence of a strong interaction between the components in the mixtures [34]. The trends observed in α^E^ values for the present mixtures suggest the formation of H-bonding between unlike molecules which is stronger in {PEG200 + [QuatGly-OEt][EtOSO_3_]} binary system. The increasing negative α^E^ values for {PEG200 + [QuatGly-OEt][EtOSO_3_]} with increase in temperature indicates more destruction of order during mixing contribute negatively to α^E^.

### 2.3. Viscosity

**Figure 5:**
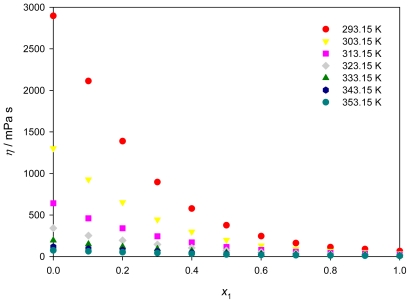
Dynamic viscosity, η, for the {PEG200 (1) + [QuatGly-OEt][EtOSO_3_] (2)} binary system as a function of mole fractions of PEG200 at different temperatures.

Knowledge of electrolyte solution viscosity is needed for the design of numerous industrial processes and provides useful insights into solution structure and interactions [35]. The measured dynamic viscosities, η, for the neat PEG200, neat [QuatGly-OEt][EtOSO_3_], and {PEG200 + [QuatGly-OEt][EtOSO_3_]} binary mixture as a function of temperature over the whole composition range are listed in Table 4. Most ionic liquids have undesirable high viscosity; one possible way to lower their viscosities is by addition of water or other diluent. Figure 5 shows the experimental dynamic viscosities for {PEG200 + [QuatGly-OEt][EtOSO_3_]} binary system, a small concentration of PEG200 significantly lowers the viscosity.

The temperature dependence becomes distinctly nonlinear, especially at high [QuatGly-OEt][EtOSO_3_] content. The Vogel-Tammann-Fulcher (VTF) equation [36,37] suitably correlates the nonlinear behavior as a function of the temperature, not only the viscosities of the neat IL but also the viscosities of the mixtures for the binary systems through the composition range.

(8) $$\eta =\eta_{o}\;\text{exp}[\frac{B}{\left(T-T_{o}\right)}]$$

where η_o_, *B*, and *T*_o_ are constants. The parameter *T*_o_ is the ideal glass transition temperature, which should be slightly below the experimental glass transition temperature *T*_g_ [38]. The adjustable parameters obtained from the VTF correlation were presented in Table 5. The obtained parameters η_o_ and *B* change smoothly with composition for binary mixtures.

The viscosities of the mixtures decrease rapidly when organic oligomers are added to the IL, this decrease is particularly evident in dilute solutions of organic oligomers in the IL. The strong coulomb interactions between the [EtOSO_3_]^−^ anion and [QuatGly-OEt]^+^ cation are weakened upon mixing with the polar organic oligomers, which leads to a higher mobility of the ions and a lower viscosity of the mixtures [39]. This indicates that the viscosity of ILs could be therefore tuned for several applications by adding an organic solvent or by changing temperature [40].

The viscosity deviations Δη (mPa·s) were calculated using:

(9) $$\Delta \eta /(\text{mPa}\cdot \text{s})=\eta -x_{1}\eta_{1}-x_{2}\eta_{2}$$

where *x*_1_ and *x*_2_ are the mole fractions of PEG200 and [QuatGly-OEt][EtOSO_3_], respectively, and η, η_1_, and η_2_ are the viscosities (mPa·s) of the solution, the pure solvent, and the pure IL, respectively.

**Figure 6:**
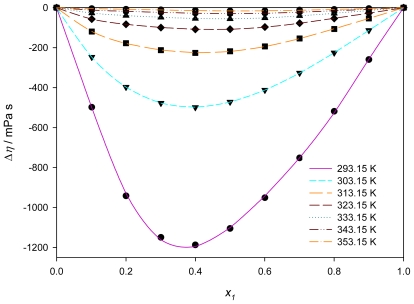
Viscosity deviations, Δη*, versus* the mole fraction at various temperatures for the binary mixture {PEG200 (1) + [QuatGly-OEt][EtOSO_3_] (2)} and fitted curves using the Redlich-Kister parameters.

Figure 6 shows the viscosity deviations as a function of the PEG200 mole fraction composition, *x*_1_, and temperature for the {PEG200 + [QuatGly-OEt][EtOSO_3_]} binary mixture. The values of viscosity deviations are compared with those obtained from the Redlich–Kister polynomial equation. The viscosity deviation for the mixture show negative deviations from ideality over the whole mole fraction range. Figure 6 also shows that the negative values of viscosity deviations decrease with increasing temperature. This can be attributed to the specific interactions in mixtures, typically H-bonds, break-up as the temperature increases [41]. The minimum value of Δη is observed at about *x*_1_ ≈ 0.4, the viscosity of a mixture depends strongly on its entropy, which is related with the liquid’s structure. Therefore, the viscosity deviation depends on molecular interactions as well as on the size and shape of the molecules [42].

### 2.4. Refractive Index Deviations and Molar Refraction Deviations

**Figure 7:**
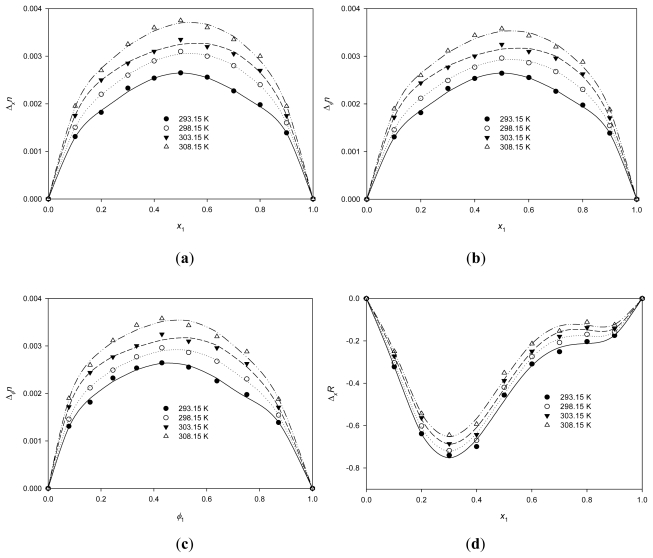

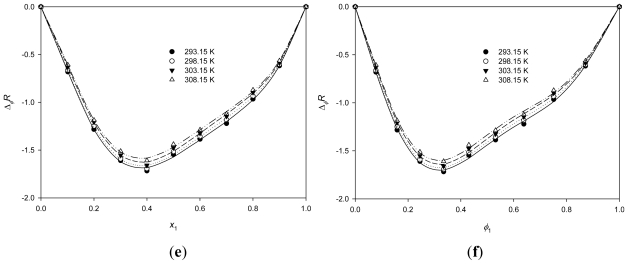
Deviation of (**a**) Δ*_x_n*; (**b**) Δ*_Ψ_n*; (**d**) Δ*_x_R* and (**e**) Δ*_Ψ_R* for the binary system {PEG200 (1) + [QuatGly-OEt][EtOSO_3_] (2)} as a function of PEG200 molar fraction, *x*_1_, at various temperatures. Deviation of (**c**) Δ*_Ψ_n* and (**f**) Δ*_Ψ_R* for the binary system {PEG200 (1) + [QuatGly-OEt][EtOSO_3_] (2)} as a function of PEG200 volume fraction, *Ψ*_1_, at various temperatures. The symbols represent experimental values, and the solid curves represent the values calculated from the Redlich-Kister parameters.

The refractive index *n* of a material is defined as the ratio *c*_o_/*c*, where *c* is the speed of light in the material and *c*_o_ is the speed of light *in vacuo*. Refractive indices *n* for the {PEG200 + [QuatGly-OEt][EtOSO_3_]} binary mixture as a function of the composition over the whole mole fraction range at *T* = 293.15 K are reported in Table 6. The refractive index deviations of molar fraction, Δ*_x_n*, from the linear additive values of the mole fractions were calculated by means of the Equation 10:

(10) $$\Delta_{x}n=n-x_{1}n_{1}-x_{2}n_{2}$$

where *n* is the refractive index of the mixture and *n*_1_ and *n*_2_ are the refractive indices of components 1 (PEG200) and 2 ([QuatGly-OEt][EtOSO_3_]), respectively. Values of Δ*_x_n* for the binary mixture are shown in Table 6 and Δ*_x_n* is plotted in Figure 7a against the mole fraction over the whole composition region.

The deviation of refractive indices Δ*_x_n* is positive over the whole range, with the maxima lying nearly at *x*_1_ ≈ 0.5. Moreover, the deviation of *n* at various volume fraction, Δ*_Ψ_n*, from ideality on volume fraction basis is given by:

(11) $$\Delta_{\Phi }n=n-\Phi_{1}n_{1}-\Phi_{2}n_{2}$$

where *Ψ*_1_ and *Ψ*_2_ are the volume fractions of component 1 (PEG200) and 2 ([QuatGly-OEt][EtOSO_3_]), respectively. Values of Δ*_Ψ_n* for the binary mixture are shown in Table 6 and Δ*_Ψ_n* is plotted in Figure 7b,c against the molar fraction and volume fraction over the whole composition region, respectively. Δ*_Ψ_n* values are symmetric against the molar fraction over the entire composition range Figure 7b, whereas Δ*_Ψ_n* are asymmetric against the volume fraction over the entire composition range Figure 7c. For the comparison of Figure 7a,b, Δ*_Ψ_n* values are similar to Δ*_x_n* and the shape of Δ*_Ψ_n* is similar to Δ*_x_n* over the entire composition range. Furthermore, the refractive index deviation increases with the increase of temperature, indicating the decrease of unlike interaction between components of the mixture.

From the experimental densities and refractive indices, the molar refractions of the IL, *R*_m_, were calculated at various temperatures using the Lorenz-Lorentz equation:

(12) $$R_{\text{m}}=\frac{n_{\text{D}}^{2}-1}{n_{\text{D}}^{2}+2}\cdot V_{\text{m}}$$

where *n*_D_ is the measured refractive index, and *V*_m_ is the molar volume of neat PEG200, neat [QuatGly-OEt][EtOSO_3_], and {PEG200 + [QuatGly-OEt][EtOSO_3_]} binary mixture. The molar refraction of the neat ILs [QuatGly-OEt][EtOSO_3_] and PEG200 are 63.9 and 48.3 at 293.15 K, respectively.

Similar representation with Δ*n* has arisen in regard to Δ*R*, the deviation of molar refraction *R* from ideality was recently noted by Fermeglia and Torriano [43]. Some papers [44,45] define it on a mole fraction basis, as

(13) $$\Delta_{x}R=R-x_{1}R_{1}-x_{2}R_{2}$$

and others [46] on a volume fraction basis, as

(14) $$\Delta_{\Phi }R=R-\Phi_{1}R_{1}-\Phi_{2}R_{2}$$

while some [43,47] report both quantities.

Values of Δ*_x_R*, Δ*_Ψ_R* for the binary mixture are shown in Table 6, and Δ*_x_R* is plotted in Figure 7d against the mole fraction over the whole composition region, whereas Δ*_Ψ_R* is plotted in Figure 7e and Figure 7f against the molar fraction and volume fraction over the whole composition region, respectively. The deviation of mole fraction Δ*_x_R* is negative over the whole range, with the minimum lying nearly at *x*_1_ ≈ 0.3, whereas Δ*_Ψ_R* values are asymmetric against both the molar fraction Figure 7e and volume fraction Figure 7f over the entire composition range. For the comparison of Figure 7d,e, the maximum of Δ*_x_R* values are about the half value of Δ*_Ψ_R* and the shape of Δ*_Ψ_R* is different to Δ*_x_R* over the entire composition range.

### 2.5. Conductivity

**Figure 8:**
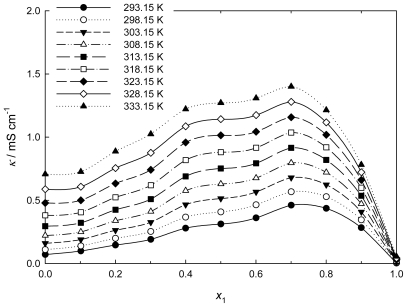
Plot of conductivity, κ, of the {PEG200 (1) + [QuatGly-OEt][EtOSO_3_] (2)} binary system *versus* mole fraction *x*_1_ at various temperatures.

The experimental data of conductivity of the neat ILs [QuatGly-OEt][EtOSO_3_], neat PEG200, and binary system are plotted against the *x*_1_ at various temperatures in Figure 8. The conductivities of neat ILs [QuatGly-OEt][EtOSO_3_], neat PEG200, and binary system at various temperatures were fitted using the VTF equation:

(15) $$\kappa =\kappa_{o}\;\text{exp}[\frac{-B^{\prime }}{\left(T-T_{o}\right)}]$$

where *T* is the absolute temperature and κ_o_^,^*B′*, and *T_o_* are adjustable parameters. The best-fit κ_o_ (mS·cm^−1^), *B′* (K), and *T*_o_ (K) parameters are given in Table 5. Neat [QuatGly-OEt][EtOSO_3_], neat PEG200, and binary system were very well fit by the VTF model over the temperature range studied.

As shown in Figure 8, it can be seen that the conductivity of the binary mixture increases with increasing amount of PEG200, goes through a maximum, and then goes down to the specific conductivity of the neat PEG200. This can be ascribed to two effects: (i) increase of viscosity and therefore reduction of the mobility of the charge carriers [48]; and (ii) reduction of the number of charge carriers due to aggregate formation [49]. The conductivity (κ) is related to the ion mobility and the number of charge carriers, which can be expressed by the following equation [48]:

(16) $$\kappa =\sum n_{i}q_{i}u_{i}$$

where *n_i_* is the number of charge carriers of species *i, q_i_* is the charge, and *u_i_* is the mobility, which is related to the mixture viscosity. It can be seen that an increase in the conductivity of a given system must be due to an increase in ion mobility and/or the number of charge carriers. Moreover, the aggregation becomes dominant at higher concentration, thus leading to a strong decrease of conductivity [49]. The maximum of conductivity is located at *x*_1_ = 0.7 and corresponds to a value of 1.4 mS·cm^−1^ at 333.15 K. It can also be observed that the conductivity increases as the temperature increases. The higher temperature may weaken the influence of aggregation to the conductivity, which suggests that a higher temperature is better for the ILs to act as electrolytes.

### 2.6. Redlich-Kister Equation for Binary System

The binary excess property (*V*_m_^E^ ) and deviations (Δη, Δ*_x_n*, Δ*_Ψ_n*, Δ*_x_R*, and Δ*_Ψ_R*) at several temperatures were fitted to a Redlich-Kister-type equation [50]:

(17) $$\Delta Y(\text{or }Y^{E})=x_{1}(1-x_{1})\sum_{i=0}^{j}A_{i}{\left(1-2x_{1}\right)}^{i}$$

where Δ*Y* (*Y*^E^) represents *V*_m_^E^ (cm^3^·mol^−1^), Δη (mPa·s), Δ*_x_n*, Δ*_Ψ_n*, Δ*_x_R*, or Δ*_Ψ_R; x*_1_ denotes the mole fraction of PEG200, *A_i_* represents the polynomial coefficients, and *j* is the degree of the polynomial expansion. The correlated results for excess molar volumes (*V*_m_^E^ ), viscosity deviations (Δη), refractive index deviations (Δ*_x_n* and Δ*_Ψ_n*), molar refraction deviations (Δ*_x_R* and Δ*_Ψ_R*), including the values of the fitting parameters *A_i_* together with the standard deviation σ, are given in Table 7, the tabulated standard deviation σ [51] is defined as:

(18) $$\sigma ={\left[\frac{\Sigma {\left(\Delta Y_{\text{exp}}-\Delta Y_{\text{cal}}\right)}^{2}}{m-n}\right]}^{1/2}$$

where *m* is the number of experimental data points and *n* is the number of estimated parameters. The subscripts “exp” and “cal” denote the values of the experimental and calculated property, respectively. As shown in Table 7, the experimentally derived *V*_m_^E^, Δη, Δ*_x_n*, Δ*_Ψ_n*, Δ*_x_R*, and Δ*_Ψ_R* values were correlated satisfactorily by the Redlich-Kister equation.

## 3. Experimental Section

### 3.1. Materials

Poly(ethylene glycol)s, diethyl sulfate, and *N*,*N*-dimethylglycine ethyl ester were obtained from commercial suppliers and used without further purification. The poly(ethylene glycol)s of average molar mass 200 was purchased from Showa Chemical Industry Co., Ltd., Japan, diethyl sulfate (99%) was purchased from Acros organics, *N*,*N*-dimethylglycine ethyl ester (>98%) was purchased from Tokyo Chemical Industry Co., Ltd.

### 3.2. 2-Ethoxy-1-ethyl-1,1-dimethyl-2-oxoethanaminium ethyl Sulfate ([QuatGly-OEt][EtOSO_3_])

Diethyl sulfate (55.5 g, 360 mmol) was added dropwise to a solution of equal molar amounts of *N*,*N*-dimethylglycine ethyl ester (47.2 g, 360 mmol) in 200 mL toluene, and then cooled in an ice-bath under nitrogen at a rate that maintained the reaction temperature below 313.15 K (highly exothermic reaction). The reaction mixture was stirred at room temperature for 1 h to 4 h. After the reaction stopped, the upper organic phase of the resulting mixture was decanted, and the lower ionic liquid phase was washed with ethyl acetate (4 × 70 mL). After the last washing, the remaining ethyl acetate was removed by rotavapor under reduced pressure. The IL obtained was dried by heating at (343.15 to 353.15) K and stirring under a high vacuum (2 × 10^−1^ Pa) for 48 h. The IL was kept in bottles under an inert gas. In order to reduce the water content to negligible values (lower than 0.03 mass %), a vacuum (2 × 10^−1^ Pa) and moderate temperature (343.15 K) were applied to the IL for several days. Yield: 83%. ^1^H NMR (300 MHz, D_2_O, ppm): 4.24 (q, 2H, O-CH_2_CH_3_), 4.19 (s, 2H, N-CH_2_COO-), 4.03 (q, 2H, O-CH_2_CH_3_), 3.56 (q, 2H, N-CH_2_CH_3_), 3.18 (s, 6H, N-CH_3_), 1.32–1.21 (m, 9H, N-CH_2_CH_3_, COOCH_2_CH_3_, CH_3_CH_2_-SO_4_). Elem. Anal. Calcd. for C_10_H_23_NO_6_S: C, 42.09%; H, 8.12%; N, 4.91%. Found: C, 42.18%; H, 8.07%; N, 4.81%.

### 3.3. Measurements

The conductivity (κ) of the ionic liquids was systematically measured with a conductivity meter (LF 340, WTW, Germany) and a standard conductivity cell (TetraCon 325, WTW, Germany). The cell constant was determined by calibration after each sample measurement using an aqueous 0.01 M KCl solution. The density of the ILs was measured with a dilatometer, which was calibrated by measuring the density of deionized water at 20, 30, 40 and 50 °C. To measure the density, IL or binary mixture was placed into the dilatometer up to the mark, sealed the top of capillary tube, which was on the top of the dilatometer, and placed into a temperature bath for 10 min to allow the temperature to equilibrate. The volume of the dilatometer is 11 cm^3^, the inside diameter of the capillary tube is 2 mm. The main interval between two marks in capillary tube is 0.1 cm^3^, and the minor interval between two marks is 0.01 cm^3^. From the correction coefficient of deionized water in capillary tube at various temperatures, we can calculate the density of neat IL or binary system by the expanded volume of liquid in capillary tube at various temperatures. Each sample was measured at least three times to determine an average value, and the values of the density are ±0.0001 g mL^−1^. The viscosity (η) of the IL was measured using a calibrated modified Ostwald viscometer (Cannon-Fenske glass capillary viscometers, CFRU, 9721-A50). The viscometer capillary diameter was 1.2 mm, as measured using a caliper (model No. PD-153) with an accuracy of ±0.02 mm. The viscometer was placed in a thermostatic water bath (TV-4000, TAMSON) whose temperature was regulated to within ±0.01 K. The flow time was measured using a stopwatch with a resolution of 0.01 s. For each sample, the experimental viscosity was obtained by averaging three to five flow time measurements. Measurements of the refractive index were conducted at 293.15 K with an ABBE refractive index instrument (Atago DR-A1), calibrated with deionized water with an accuracy greater than ±2 × 10^−4^. The water content of synthesized IL [QuatGly-OEt][EtOSO_3_] was determined using the Karl-Fischer method; the content was below 100 ppm. The nuclear magnetic resonance (NMR) spectra of the synthesized ILs were recorded on a BRUKER AV300 spectrometer and calibrated with tetramethylsilane (TMS) as the internal reference.

## 4. Conclusions

This paper is a systematic characterization of physical properties for IL binary system. Experimental density, refractive index, and dynamic viscosity data for (PEG200 + [QuatGly-OEt][EtOSO_3_]) binary system are presented as a function of the temperature at atmospheric pressure. The viscosity and conductivity of pure IL and (PEG200 + [QuatGly-OEt][EtOSO_3_]) binary system at different temperatures are fitted using the Vogel-Tammann-Fulcher (VTF) equation with good accuracy. The excess molar volumes, excess volume expansivity, viscosity deviations, and molar refraction deviations show negative deviations from the ideal solution. Whereas the deviation of the refractive index has a positive value over the whole composition range, the results are interpreted in terms of molecular interactions in the (PEG200 + [QuatGly-OEt][EtOSO_3_]) binary mixture. The conductivity increases to a maximum value when the concentration of PEG200 is increased and decreases after the maximum. The data obtained will be helpful for the application of the ionic liquids as electrolytes and also useful for the ionic liquids database. Moreover, the present data on density and excess molar volumes will be useful for the efficient design of gas-liquid contacting equipment for commercial applications.

## Figures and Tables

**Table 1:** Experimental density (ρ) and excess molar volume (*V*_m_^E^ ) for the binary system {PEG200 (1) + [QuatGly-OEt][EtOSO_3_] (2)}.

	*T/*K												
x_1_	293.15	298.15	303.15	308.15	313.15	318.15	323.15	328.15	333.15	338.15	343.15	348.15	353.15
	ρ/g cm^−3^												
0	1.2191	1.2160	1.2128	1.2097	1.2066	1.2035	1.2004	1.1973	1.1943	1.1913	1.1882	1.1852	1.1822
0.1015	1.2215	1.2183	1.2151	1.2119	1.2087	1.2055	1.2024	1.1993	1.1961	1.1930	1.1900	1.1869	1.1839
0.2000	1.2221	1.2189	1.2157	1.2125	1.2094	1.2062	1.2030	1.1998	1.1966	1.1934	1.1902	1.1871	1.1839
0.3007	1.2180	1.2148	1.2115	1.2083	1.2051	1.2018	1.1986	1.1954	1.1921	1.1889	1.1857	1.1824	1.1792
0.3999	1.2100	1.2066	1.2033	1.1999	1.1966	1.1933	1.1900	1.1867	1.1835	1.1802	1.1770	1.1738	1.1706
0.5000	1.1969	1.1935	1.1902	1.1869	1.1836	1.1802	1.1769	1.1736	1.1703	1.1669	1.1636	1.1603	1.1570
0.6004	1.1847	1.1813	1.1779	1.1746	1.1712	1.1678	1.1644	1.1610	1.1576	1.1542	1.1508	1.1475	1.1441
0.7003	1.1735	1.1700	1.1666	1.1631	1.1597	1.1562	1.1527	1.1493	1.1458	1.1424	1.1389	1.1355	1.1320
0.8002	1.1619	1.1582	1.1546	1.1510	1.1474	1.1438	1.1402	1.1367	1.1332	1.1297	1.1262	1.1228	1.1194
0.8999	1.1495	1.1457	1.1419	1.1382	1.1345	1.1308	1.1271	1.1235	1.1199	1.1163	1.1127	1.1092	1.1056
1	1.1312	1.1273	1.1234	1.1195	1.1157	1.1119	1.1081	1.1044	1.1007	1.0970	1.0933	1.0896	1.0860
	*V*_m_^E^ /cm^3^ mol^−1^												
0	0.0000	0.0000	0.0000	0.0000	0.0000	0.0000	0.0000	0.0000	0.0000	0.0000	0.0000	0.0000	0.0000
0.1015	−1.7372	−1.7458	−1.7543	−1.7629	−1.7714	−1.7799	−1.7885	−1.7970	−1.8056	−1.8141	−1.8226	−1.8312	−1.8397
0.2000	−3.0868	−3.1224	−3.1551	−3.1847	−3.2112	−3.2347	−3.2551	−3.2723	−3.2864	−3.2974	−3.3051	−3.3096	−3.3108
0.3007	−3.6416	−3.6832	−3.7219	−3.7574	−3.7899	−3.8193	−3.8455	−3.8686	−3.8885	−3.9051	−3.9186	−3.9287	−3.9356
0.3999	−3.5474	−3.5758	−3.6042	−3.6326	−3.6610	−3.6895	−3.7179	−3.7463	−3.7747	−3.8031	−3.8315	−3.8599	−3.8883
0.5000	−2.6761	−2.7217	−2.7640	−2.8032	−2.8392	−2.8719	−2.9014	−2.9276	−2.9505	−2.9700	−2.9861	−2.9988	−3.0081
0.6004	−2.0795	−2.1240	−2.1653	−2.2032	−2.2379	−2.2692	−2.2971	−2.3216	−2.3427	−2.3603	−2.3745	−2.3851	−2.3922
0.7003	−1.7384	−1.7815	−1.8213	−1.8577	−1.8906	−1.9201	−1.9461	−1.9685	−1.9874	−2.0027	−2.0144	−2.0225	−2.0268
0.8002	−1.4389	−1.4592	−1.4795	−1.4998	−1.5202	−1.5405	−1.5608	−1.5811	−1.6014	−1.6217	−1.6420	−1.6623	−1.6826
0.8999	−1.1124	−1.1252	−1.1380	−1.1508	−1.1636	−1.1764	−1.1892	−1.2020	−1.2148	−1.2276	−1.2404	−1.2532	−1.2660
1	0.0000	0.0000	0.0000	0.0000	0.0000	0.0000	0.0000	0.0000	0.0000	0.0000	0.0000	0.0000	0.0000

**Table 2:** The adjustable parameters of density (ρ = *b*_0_ + *b*_1_·*T* + *b*_2_·*T*_2_) at various temperatures for neat PEG200 (*x*_1_ = 1), [QuatGly-OEt][EtOSO_3_] (*x*_1_ = 0), and binary system {PEG200 (1) + [QuatGly-OEt][EtOSO_3_] (2)}.

*x*_1_	*b*_0_	10^4^*b*_1_	10^7^*b*_2_	10^4^ σ
0	1.432	−8.2	3.15	1.6
0.1015	1.439	−8.4	3.27	3.6
0.2000	1.409	−6.4	0	1.7
0.3007	1.408	−6.5	0	3.2
0.3999	1.44	−8.9	3.62	2.0
0.5000	1.392	−6.7	0	1.9
0.6004	1.383	−6.8	0	1.7
0.7003	1.376	−6.9	0	3.6
0.8002	1.415	−9.9	4.41	4.1
0.8999	1.413	−10.4	4.74	7.1
1	1.405	−10.8	5.11	12.2

**Table 3:** Experimental volume expansivity (α) and the excess volume expansivity (α^E^) for the binary system {PEG200 (1) + [QuatGly-OEt][EtOSO_3_] (2)}.

x_1_	*T/*K												
	293.15	298.15	303.15	308.15	313.15	318.15	323.15	328.15	333.15	338.15	343.15	348.15	353.15
	10^4^ α/K^−1^												
0	5.0413	5.0544	5.0675	5.0807	5.0938	5.1069	5.1200	5.1331	5.1462	5.1593	5.1724	5.1855	5.1986
0.1015	5.1363	5.1499	5.1635	5.1771	5.1907	5.2044	5.2180	5.2316	5.2452	5.2588	5.2724	5.2860	5.2996
0.2000	5.2124	5.2260	5.2397	5.2534	5.2673	5.2812	5.2952	5.3092	5.3233	5.3375	5.3518	5.3662	5.3806
0.3007	5.3144	5.3285	5.3428	5.3571	5.3715	5.3859	5.4005	5.4151	5.4298	5.4446	5.4594	5.4744	5.4894
0.3999	5.4207	5.4358	5.4510	5.4662	5.4814	5.4966	5.5118	5.5270	5.5422	5.5573	5.5725	5.5877	5.6029
0.5000	5.5578	5.5733	5.5889	5.6046	5.6203	5.6361	5.6521	5.6681	5.6842	5.7004	5.7167	5.7331	5.7496
0.6004	5.7170	5.7334	5.7499	5.7665	5.7831	5.7999	5.8168	5.8337	5.8508	5.8680	5.8852	5.9026	5.9201
0.7003	5.8952	5.9126	5.9301	5.9478	5.9655	5.9834	6.0013	6.0194	6.0376	6.0558	6.0742	6.0927	6.1114
0.8002	6.1003	6.1196	6.1389	6.1582	6.1776	6.1969	6.2162	6.2355	6.2548	6.2741	6.2935	6.3128	6.3321
0.8999	6.3560	6.3770	6.3980	6.4190	6.4400	6.4610	6.4820	6.5030	6.5240	6.5450	6.5660	6.5870	6.6080
1	6.6532	6.6763	6.6993	6.7224	6.7455	6.7685	6.7916	6.8146	6.8377	6.8607	6.8838	6.9069	6.9299
	10^4^ α^E^/K^−1^												
0	0.0000	0.0000	0.0000	0.0000	0.0000	0.0000	0.0000	0.0000	0.0000	0.0000	0.0000	0.0000	0.0000
0.1015	−0.0318	−0.0322	−0.0325	−0.0329	−0.0333	−0.0337	−0.0340	−0.0344	−0.0348	−0.0352	−0.0356	−0.0360	−0.0363
0.2000	−0.0850	−0.0863	−0.0875	−0.0886	−0.0896	−0.0906	−0.0915	−0.0923	−0.0930	−0.0937	−0.0943	−0.0948	−0.0953
0.3007	−0.1221	−0.1237	−0.1253	−0.1268	−0.1282	−0.1296	−0.1308	−0.1320	−0.1331	−0.1342	−0.1351	−0.1360	−0.1368
0.3999	−0.1604	−0.1620	−0.1635	−0.1651	−0.1667	−0.1682	−0.1698	−0.1714	−0.1729	−0.1745	−0.1761	−0.1777	−0.1793
0.5000	−0.1771	−0.1794	−0.1815	−0.1836	−0.1856	−0.1875	−0.1893	−0.1910	−0.1926	−0.1942	−0.1956	−0.1970	−0.1983
0.6004	−0.1812	−0.1835	−0.1858	−0.1879	−0.1900	−0.1920	−0.1939	−0.1957	−0.1974	−0.1990	−0.2004	−0.2018	−0.2031
0.7003	−0.1751	−0.1775	−0.1797	−0.1819	−0.1839	−0.1859	−0.1877	−0.1894	−0.1911	−0.1926	−0.1940	−0.1953	−0.1965
0.8002	−0.1524	−0.1540	−0.1555	−0.1570	−0.1585	−0.1601	−0.1616	−0.1632	−0.1647	−0.1662	−0.1678	−0.1693	−0.1709
0.8999	−0.0904	−0.0913	−0.0922	−0.0932	−0.0941	−0.0950	−0.0960	−0.0969	−0.0979	−0.0988	−0.0997	−0.1007	−0.1016
1	0.0000	0.0000	0.0000	0.0000	0.0000	0.0000	0.0000	0.0000	0.0000	0.0000	0.0000	0.0000	0.0000

**Table 4:** Experimental dynamic viscosity (η) and viscosity deviation (Δη) for the binary system {PEG200 (1) + [QuatGly-OEt][EtOSO_3_] (2)}.

x_1_	*T/*K												
	293.15	298.15	303.15	308.15	313.15	318.15	323.15	328.15	333.15	338.15	343.15	348.15	353.15
	η/mPa·s												
0	2897.3	1917.3	1301.5	904.2	641.6	464.2	341.8	255.9	194.4	149.8	116.9	92.4	73.8
0.1015	2111.9	1374.7	926.5	644.0	460.0	336.7	251.9	192.1	149.2	117.7	94.2	76.4	62.7
0.2000	1389.2	937.5	652.0	465.9	341.0	255.0	194.4	150.8	118.9	95.1	77.0	63.2	52.4
0.3007	896.4	622.9	445.2	326.1	244.3	186.7	145.2	114.8	92.1	74.9	61.7	51.3	43.2
0.3999	577.5	409.6	298.4	222.6	169.6	131.7	104.0	83.4	67.9	55.9	46.6	39.2	33.3
0.5000	376.6	270.5	199.4	150.4	115.8	90.9	72.5	58.7	48.1	40.0	33.6	28.5	24.4
0.6004	246.3	178.1	132.6	101.2	79.0	62.8	50.9	41.8	34.9	29.4	25.1	21.6	18.8
0.7003	162.3	120.3	91.6	71.5	57.0	46.2	38.1	31.9	27.0	23.2	20.1	17.6	15.5
0.8002	112.9	85.8	66.8	53.0	42.8	35.1	29.2	24.7	21.0	18.1	15.7	13.8	12.2
0.8999	89.8	68.5	53.4	42.5	34.5	28.4	23.7	20.0	17.1	14.8	12.9	11.3	10.1
1	65.8	51.0	40.3	32.4	26.4	21.9	18.3	15.5	13.2	11.4	9.9	8.7	7.7
	Δη/mPa·s												
0	0.0	0.0	0.0	0.0	0.0	0.0	0.0	0.0	0.0	0.0	0.0	0.0	0.0
0.1015	−498.0	−353.2	−247.0	−171.7	−119.1	−82.6	−57.1	−39.3	−26.8	−18.1	−11.9	−7.4	−4.3
0.2000	−941.7	−606.6	−397.3	−264.0	−177.6	−120.7	−82.7	−57.0	−39.3	−27.1	−18.5	−12.5	−8.2
0.3007	−1149.5	−733.3	−477.2	−316.0	−212.3	−144.5	−99.3	−68.7	−47.8	−33.3	−23.1	−15.9	−10.7
0.3999	−1187.4	−761.4	−498.8	−333.0	−226.0	−155.6	−108.4	−76.3	−54.1	−38.6	−27.6	−19.7	−14.0
0.5000	−1104.9	−713.7	−471.6	−317.9	−218.2	−152.1	−107.6	−77.0	−55.7	−40.6	−29.8	−22.0	−16.3
0.6004	−951.0	−618.8	−411.8	−279.6	−193.3	−135.8	−96.7	−69.7	−50.8	−37.3	−27.6	−20.5	−15.3
0.7003	−752.0	−490.0	−326.6	−222.2	−153.8	−108.2	−77.1	−55.6	−40.5	−29.7	−21.9	−16.2	−12.0
0.8002	−518.7	−338.1	−225.6	−153.6	−106.5	−75.1	−53.7	−38.9	−28.4	−21.0	−15.6	−11.6	−8.7
0.8999	−259.5	−169.4	−113.1	−77.1	−53.5	−37.7	−27.0	−19.5	−14.2	−10.5	−7.8	−5.8	−4.3
1	0.0	0.0	0.0	0.0	0.0	0.0	0.0	0.0	0.0	0.0	0.0	0.0	0.0

**Table 5:** The VTF equation parameters of viscosity ( $\eta^{-1}=\eta_{o}\;\text{exp}[\frac{-B}{\left(T-T_{o}\right)}]$) and conductivity ( $\kappa =\kappa_{o}\;\text{exp}[\frac{-B^{\prime }}{\left(T-T_{o}\right)}]$) for neat PEG200 (*x*_1_ = 1), [QuatGly-OEt][EtOSO_3_] (*x*_1_ = 0), and binary system {PEG200 (1) + [QuatGly-OEt][EtOSO_3_] (2)}.

	η				κ			
	
*x*_1_	η_o_ (mPa·s)	*T*_o_ (K)	*B* (K)	*R*^2^^a^	κ_o_ (mS·cm^−1^)	*T*_o_ (K)	*B* (K)	*R*^2^^a^
0	0.24	190.3	967.8	0.999	24.9	230.7	365.5	0.999
0.1015	0.282	193.4	888	0.999	52.2	206.9	540.4	0.999
0.2000	0.295	194.4	836.9	0.999	43.6	206.6	493.1	0.999
0.3007	0.292	193.8	800.9	0.999	53.3	198.5	533.3	0.999
0.3999	0.277	191.2	780.7	0.999	16.2	223.7	281.9	0.999
0.5000	0.263	187.4	764.5	0.999	11.3	231.3	222.1	0.999
0.6004	0.204	179.9	795.2	0.999	10.0	229.1	212.5	0.999
0.7003	0.129	172.7	856.9	0.999	8.7	227.5	192.8	0.999
0.8002	0.102	176.2	831	0.999	6.6	227.2	178.9	0.999
0.8999	0.123	186	718.2	0.999	3.4	235.1	144.6	0.999
1	0.471	198.5	455.6	0.999	25.6	202.3	818.1	0.999

a Correlation coefficient.

**Table 6:** The refractive index *n*, Δ*_x_n*, Δ*_Ψ_n*, Δ*_x_R*, and Δ*_Ψ_R* for the binary mixture of {PEG200 (1) + [QuatGly-OEt][EtOSO_3_] (2)} at 293.15 K (*n* ± 0.0003).

*x*_1_	*n*	10^3^*Δ_x_n*	10^3^*Δ_Ψ_n*	*Δ_x_R*	*Δ_Ψ_R*
0	1.4586	0	0	0	0
0.1015	1.4599	1.310	1.308	−0.3224	−0.6806
0.2000	1.4604	1.820	1.816	−0.6385	−1.2827
0.3007	1.4609	2.330	2.325	−0.7403	−1.6094
0.3999	1.4611	2.540	2.533	−0.6990	−1.7176
0.5000	1.4612	2.650	2.643	−0.4559	−1.5466
0.6004	1.4611	2.560	2.553	−0.3091	−1.3859
0.7003	1.4608	2.270	2.264	−0.2514	−1.2212
0.8002	1.4605	1.980	1.975	−0.2036	−0.9649
0.8999	1.4599	1.390	1.387	−0.1746	−0.6169
1	1.4585	0	0	0	0

**Table 7:** Redlick–Kister fitting coefficients *A_k_* and the standard deviation σ of the *V*^E^, Δη, Δ*_x_n*, Δ*_Ψ_n*, Δ*_x_R*, and Δ*_Ψ_R* for the binary mixture of {PEG200 (1) + [QuatGly-OEt][EtOSO_3_] (2)} system.

*T/*K	*A*_0_	*A*_1_	*A*_2_	*A*_3_	*A*_4_	σ
	*V*^E^/cm^3^ mol^−1^					
293.15	−11.107	−14.337	−10.953	15.942	6.0212	0.087333
298.15	−11.271	−14.371	−11.168	16.012	6.4747	0.082121
303.15	−11.425	−14.401	−11.355	16.083	6.8586	0.078529
308.15	−11.569	−14.429	−11.511	16.156	7.1722	0.076233
313.15	−11.704	−14.455	−11.638	16.229	7.4151	0.074909
318.15	−11.83	−14.477	−11.734	16.304	7.5866	0.074295
323.15	−11.946	−14.285	−11.801	16.379	7.6863	0.077588
328.15	−12.052	−14.514	−11.836	16.456	7.7134	0.074634
333.15	−12.149	−14.529	−11.841	16.535	7.6676	0.075618
338.15	−12.236	−14.54	−11.815	16.614	7.548	0.077371
343.15	−12.313	−14.549	−11.757	16.695	7.3543	0.080197
348.15	−12.38	−14.555	−11.668	16.777	7.0857	0.084452
353.15	−12.436	−14.558	−11.547	16.861	6.7417	0.090499
	Δη/mPa·s					
293.15	−4402.7	−2700.8	−1378.1	1669.7	2724.6	11.464135
298.15	−2852.6	−1531.9	−553.24	438.22	803.55	2.679943
303.15	−1888.4	−885.33	−175.33	−36	41.466	0.836423
308.15	−1274.4	−517.5	−5.2237	−192.58	−228.25	1.451973
313.15	−874.81	−303.39	66.785	−219.52	−292.5	1.395598
318.15	−609.61	−176.48	92.393	−197.67	−276.01	1.101902
323.15	−430.41	−100.24	96.438	−161.37	−232.64	0.794003
328.15	−308.33	−64.397	117.64	−82.435	−258.8	1.295174
333.15	−221.49	−26.014	81.89	−92.312	−141.62	0.448191
338.15	−160.83	−9.0716	71.941	−65.834	−105.27	0.431571
343.15	−117.42	1.0083	62.409	−44.859	−76.01	0.461003
348.15	−86.004	6.8152	53.81	−28.597	−53.013	0.496660
353.15	−63.043	9.9539	46.291	−16.169	−35.213	0.523728
	Δ*_x_n*					
293.15	1.06 × 10^−2^	−0.06 × 10^−3^	−0.4 × 10^−3^	−1 × 10^−3^	11 × 10^−3^	0.485 × 10^−4^
298.15	1.22 × 10^−2^	−1.3 × 10^−3^	3.3 × 10^−3^	0.8 × 10^−3^	6.9 × 10^−3^	0.304 × 10^−4^
303.15	1.3 × 10^−2^	−1.7 × 10^−3^	6.8 × 10^−3^	2.4 × 10^−3^	5.1 × 10^−3^	0.688 × 10^−4^
308.15	1.48 × 10^−2^	−1.5 × 10^−3^	4.7 × 10^−3^	1.8 × 10^−3^	9.1 × 10^−3^	0.845 × 10^−4^
	Δ*_Ψ_n* against *x*_1_					
293.15	1.06 × 10^−2^	−0.055 × 10^−3^	−0.4 × 10^−3^	−1 × 10^−3^	11 × 10^−3^	0.469 × 10^−4^
298.15	1.17 × 10^−2^	−1.2 × 10^−3^	3.3 × 10^−3^	0.8 × 10^−3^	6.9 × 10^−3^	0.312 × 10^−4^
303.15	1.26 × 10^−2^	−1.6 × 10^−3^	6.7 × 10^−3^	2.4 × 10^−3^	5.1 × 10^−3^	0.686 × 10^−4^
308.15	1.41 × 10^−2^	−1.4 × 10^−3^	4.7 × 10^−3^	1.8 × 10^−3^	9.1 × 10^−3^	0.847 × 10^−4^
	Δ*_Ψ_n* against *Ψ*_1_					
293.15	1.05 × 10^−2^	1.8 × 10^−3^	−0.2 × 10^−3^	0.2 × 10^−3^	11.5 × 10^−3^	0.73 × 10^−4^
298.15	1.17 × 10^−2^	0.5 × 10^−3^	1.9 × 10^−3^	3.3 × 10^−3^	9.2 × 10^−3^	0.49 × 10^−4^
303.15	1.27 × 10^−2^	−0.4 × 10^−3^	4.2 × 10^−3^	6.4 × 10^−3^	8.7 × 10^−3^	0.85 × 10^−4^
308.15	1.42 × 10^−2^	0.4 × 10^−3^	2.9 × 10^−3^	5.3 × 10^−3^	12 × 10^−3^	1.19 × 10^−4^
	Δ*_x_R*					
293.15	−1.9293	−3.8038	−3.0402	4.3952	2.8158	0.025
298.15	−1.8058	−3.8961	−2.6609	4.5773	2.4094	0.0255
303.15	−1.7031	−3.9151	−2.306	4.7678	2.2485	0.0281
308.15	−1.5286	−3.8653	−2.5239	4.7144	2.6929	0.025
	Δ*_Ψ_R* against *x*_1_					
293.15	−6.2921	−3.1958	−3.1249	4.4072	2.8141	0.025
298.15	−6.192	−3.2867	−2.7455	4.5892	2.4077	0.0254
303.15	−6.0536	−3.3125	−2.3895	4.7795	2.2468	0.0281
308.15	−5.8814	−3.2642	−2.6069	4.7261	2.6912	0.025
	Δ*_Ψ_R* against *Ψ*_1_					
293.15	−5.7634	−3.9989	−6.3897	2.2381	5.7545	0.0279
298.15	−5.6501	−4.1099	−6.1522	2.5598	5.5137	0.0271
303.15	−5.5082	−4.1715	−5.9053	2.9193	5.5117	0.0306
308.15	−5.3499	−4.0698	−6.0323	2.8287	5.8762	0.031
